# Additive manufacturing of micrometric crystallization vessels and single crystals

**DOI:** 10.1038/srep36786

**Published:** 2016-11-10

**Authors:** Oded Halevi, Hui Jiang, Christian Kloc, Shlomo Magdassi

**Affiliations:** 1 Casali Center of Applied Chemistry, Institute of Chemistry, The Hebrew University of Jerusalem, Jerusalem 91904, Israel; 2 School of Materials Science and Engineering, Nanyang Technological University, 639798, Singapore

## Abstract

We present an all-additive manufacturing method that is performed at mild conditions, for the formation of organic single crystals at specific locations, without any photolithography prefabrication process. The method is composed of two steps; inkjet printing of a confinement frame, composed of a water soluble electrolyte. Then, an organic semiconductor solution is printed within the confinement to form a nucleus at a specific location, followed by additional printing, which led to the growth of a single crystal. The specific geometry of the confinement enables control of the specific locations of the single crystals, while separating the nucleation and crystal growth processes. By this method, we printed single crystals of perylene, which are suitable for the formation of OFETs. Moreover, since this method is based on a simple and controllable wet deposition process, it enables formation of arrays of single crystals at specific locations, which is a prerequisite for mass production of active organic elements on flexible substrates.

Additive manufacturing processes such as inkjet and 3D printing are rapidly entering into new fields, bringing new approaches to what many relate to as the third industrial revolution. Among the benefits and advantages of these technologies is the production of tailor-made tools and even reaction vessels^1^,^2^. These items and devices can be designed for specific functions and purposes such as pressure durable reactors, incorporation of catalysts within reactor walls^3^, and formation of electronically active devices^4^,^5^.

Additive manufacturing can be highly efficient when combined with the technology of organic semiconductors. These materials have become a major area of research due to the maturity of devices based on them; such as organic light-emitting diodes (OLEDs), which are used for active-matrix OLED TV, or polymer LEDs for lighting. Therefore, intense efforts have led to the optimization of organic semiconductors technologies and discoveries of new materials^6^,^7^. These compounds demonstrate significant differences compared with inorganic-based electronic materials and devices; low manufacturing costs, solubility, flexibility, and simple processing methods are some of the qualities that make these materials so promising for utilization in various technologies^8^. Due to these differences, organic semiconductors seem to be preferred for low-energy manufacturing technologies like printing, and for instruments in which the space or element dimensions are crucial, such as printed electronics on the back of an active-matrix OLED TV screen. However, printing elements in the micrometers scale requires different materials properties than those designated for photolithographic based technologies and for the fabrication of electronic circuits in the nanometers scale. The most important issues are the physical properties of materials that would enable current conduction in long paths and efficient switching of such large devices. Therefore, due to the requirement of a well-controlled structure and quality of the electronic active parts of circuits, organic semiconductors would be preferred if made from small, high quality organic single crystals.

Polycyclic aromatic hydrocarbons (PAHs) such as perylene, rubrene, anthracene and their derivatives demonstrate semiconducting behavior due to their conjugated π-electrons system, and are used in many electronic devices such as organic field-effect transistors (OFETs)^9^. The highest electronic performances, are mostly obtained in devices that are based on single crystals, which demonstrate high purity, short and long-term structural order, and lack of grain boundaries^10^,^11^. However, until recently, the fabrication of such devices required the growth of crystals from solution or by physical vapor deposition (PVD)^12^. These two methods limit the applicability of single crystal based devices due to time consuming and crystal transferring difficulties; thus, the controlled and fast deposition of single crystals of these materials has become a challenging research area. In order to gain significant control over the growth of a large number of identical single crystals, forming active elements of electronic circuits, it is required to create a single nucleus at a specific location. Once a nucleus is formed, additional building blocks should be supplied to the nucleus for its growth. Some efforts have been made to gain control over the deposition of both organic and inorganic single crystals^13^,^14^,^15^,^16^. A highly effective approach to control the assembly and organization of functional materials in general, and specifically their formation of single crystals, is by spatial confinement. The importance of spatial confinement is due to the ability to control various features such as crystal orientation, location, the mean size of crystalline domains, and the ease of fabrication^17^. This was demonstrated for example by Moto *et al*., where individual single crystals were grown by the deposition of solution into a defined confinement frame that enabled geometrical separation between nucleation control region (NCR) and growth control region (GCR)^18^. Another case in which spatial confinement enabled the formation of defined structures as presented by Valle *et al*., who deposited laminin on highly hydrophobic, anti-adhesive substrates such as Teflon-AF by lithographically controlled wetting, in order to control cell adhesion and growth^19^. This deposition method has been also utilized by Gentili *et al*. to grow micrometric single crystals at specific locations by confining the solution of an organic semiconductor^20^.

However, in the field of crystallization, there are no reports in which only additive manufacturing technologies, such as inkjet printing, are utilized for the process of crystal growth. Inkjet printing is a powerful additive tool, which enables the deposition of many forms of liquid inks on a variety of substrates. It is a fast, cost-effective, highly precise and controllable method with a micrometric resolution^21^,^22^. Hasegawa *et al*. used a substrate with a pre-fabricated hydrophilic pattern, which was prepared by photolithography^23^, and utilized inkjet printing for the formation of single crystals at specific location. In their work, they inkjet printed an anti-solvent, followed by printing a semiconductor solution, thus resulting in crystal formation. Later, Park *et al*. used source and drain electrodes, which were made by PVD as nucleation sites, while depositing an organic solution into a confined hydrophilic area by inkjet printing^24^. The hydrophilic areas were also made by a photolithographic process, which was performed prior to printing. These methods enabled the formation of pure, micrometric single crystals, which were suitable for fabrication of high-performance devices such as OFETs. However, these and other methods require the combination of various fabrication and deposition processes, which mostly include photolithography, lift-off techniques, and solution deposition methods^14^,^16^,^25^. So far, no one has presented an all-additive manufacturing approach for the fabrication of the crystallization areas, followed by formation of single crystals also by deposition of the organic solution. This approach for the controlled growth of single crystals has many implications concerning the time and cost of the process and also its applicability to a variety of materials, substrates including plastics, at large production scale. Lately, inkjet printing has been used to etch microwells and to deposit a solution of an organic semiconductor^26^. However, this method enabled forming polycrystalline film in each microwell and not a single crystal.

Herein, we propose an additive manufacturing process, which is based on inkjet-printing for the controlled growth of **single crystals** at specific locations on the substrate. The proposed method utilizes the printer both as a high-resolution lithographic tool for the pre-fabrication of a complex confinement frame, in which the crystallization process takes place; and for the deposition of the semiconductor solution in a controlled and gradual manner, which enables separation of nucleation from growth. By this method, we printed single crystals of perylene, which are suitable for the formation of OFETs. Moreover, since this method is in essence a one-step wet deposition process it enables formation of arrays of single crystals at specific locations, which is a prerequisite for mass production processes of active organic elements on flexible substrates.

## Results

### Inkjet-printing of confinement frames

**Figure 1:**
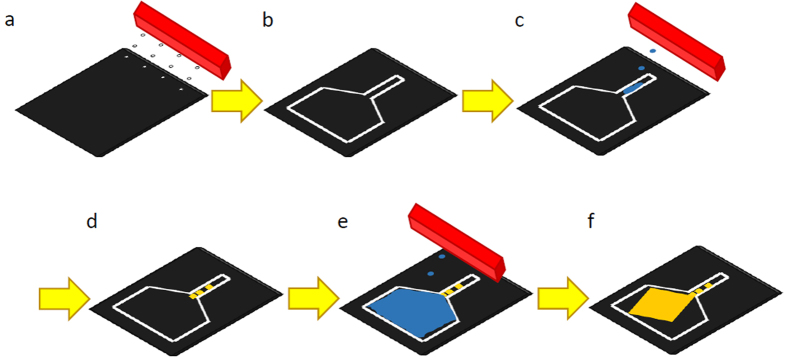
The method of inkjet printing of a single crystal. First, AlCl_3_ confinement frame is printed in the desired funnel-like shape (steps **a,b**). Then, several drops are printed into the narrow part of the frame that acts as the NCR (step **c**). As the solution evaporates, microcrystals are formed (step d). Following this, the solution is printed into the GCR, until it reaches the microcrystal (step **e**). The growth of a single crystal commences at the dividing line of the two regions (step **f**).

The printing of confined frames was the first step towards obtaining a large number of individual micrometric crystals at specific location on surfaces that are as large as A4 sheet. In this method (Fig. 1), each frame would act as a micrometric crystallization vessel for the growth of a single crystal of perylene. Confinement of solutions can be achieved by inducing mechanical barrier due to a presence of solid walls, or by causing hydrophilic/lipophilic interactions.

**Figure 2:**
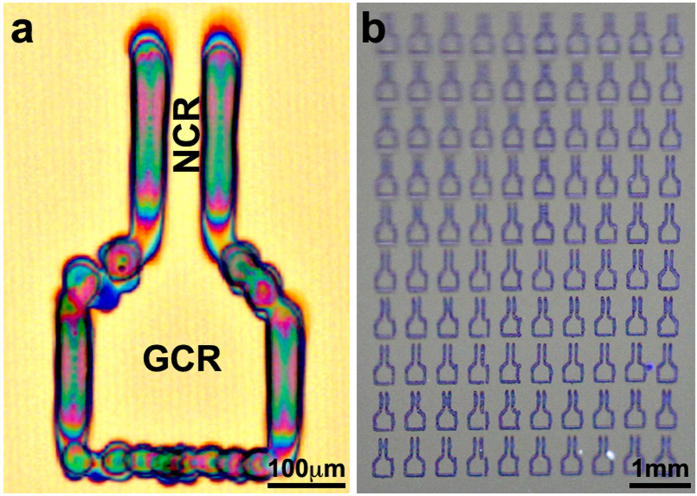
Inkjet-printed AlCl_3_ confinement frames on SiO_2_/Si. (**a**) A single frame, height of 325 ± 41 nm. (**b**) An array of AlCl_3_ frames.

In our approach we use both; printing of a hydrophilic frame that also acts as a mechanical barrier, while having a unique design, with pre-determined wall height and thickness. The building block of the wall is AlCl_3_, which easily dissolves in water at relatively high concentrations, thus enables a sufficient load of the wall forming material. In addition, it does not precipitate too rapidly and adsorbs water from the air, thus minimizing typical print-head clogging problems during printing. Moreover, upon drying, the precipitated building block is a hydrated salt, therefore presenting hydrophilic characteristics that will enable confinement of organic solutions without the dissolution of the wall material. An AlCl_3_ solution in a 1:1 mixture of diethylene glycol butyl ether (DB) and deionized water was inkjet-printed onto a silicon wafer, to fabricate defined micrometric geometrical frames, as shown in Fig. 2a. The figure demonstrates a typical printed single micrometric crystallization frame with a specific design that will be explained later, and arrays of many such frames, which can be utilized for making arrays of single crystals as will be presented in the following sections. Each frame was fabricated by printing five layers of the electrolyte. The height of each wall of the frame is about 300 nanometers, and its width is about 60 micrometers. Obviously, these dimensions can be modified by changing the printing parameters and the physicochemical properties of the printing solution.

### Controlled crystallization of single crystals

Following the printing of the confinement frames, the organic solution of the semiconductor was printed within the frames, to form a single crystal upon evaporation of the solvent. The chosen “printing ink” in this work was a concentrated solution of perylene in a 1:1 mixture of isophorone and propylene glycol phenylether (PPh). Although not considered a relatively high-performance organic semiconductor on its own, perylene was chosen due to its being a parent compound for many semiconducting materials^27^. Since we aimed to initiate the crystallization process at a specific site, we printed and tested confinement frames with a variety of geometrical shapes and sizes (see Supplementary Fig. S1). All frames were composed of a nucleation controlled region (NCR) connected to a growth controlled region (GCR). Figure 2a shows the optimal geometry of the printed frame. This structure was selected for two reasons: Firstly, to obtain a better separation between growth and nucleation processes, and secondly, to direct the flow of the solution towards the formed nucleus. The latter should minimize the dependency on the diffusion rate of perylene molecules in the solution towards the forming crystal, and lower the chances of nucleation and growth at undesired locations in the growth region. In this funnel-like shape of the frame, the NCR is narrower compared to the GCR, causing a capillary flow of the solution from the GCR towards the NCR.

**Figure 3:**
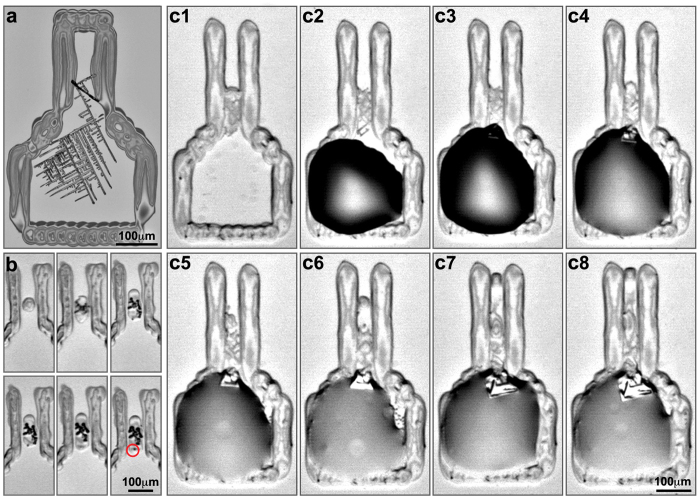
The growth process of a crystal. (**a**) Multi-crystalline needles of perylene, formed due to the absence of a single nucleus. (**b**) Formation of a nucleus in the NCR. Marked with a red circle is the microcrystal that reached the NCR-GCR dividing line and was later used as a nucleus for crystal growth. (**c**) Gradual growth of a crystal in the dividing line of the NCR and the GCR: Ink is deposited in the GCR and gradually spreads toward the microcrystal located in the NCR. Following is the growth of the single crystal in the GCR. (c2-c8) The directional flow of the solution from the GCR to the NCR is shown as the solution moves from the GCR to the edge of the NCR over time.

Initial experiments on SiO_2_/Si and polyethylene terephthalate (PET) substrates showed that the perylene solution, which was printed into the GCR, did not spread sufficiently fast into the NCR. Thus, upon solvent evaporation, instead of forming a single nucleus, several small needle-like perylene crystals were observed (Fig. 3a). Therefore, an active approach of forming a single nucleus was necessary. This was achieved by gradual deposition of the organic solution at a specific location in the NCR by precise printing of single droplets, enabling the formation of a nucleus close to the GCR (Fig. 3b). In the majority of cases, a thick line of microcrystals were formed along the NCR, due to the spreading of the deposited solution along the walls of the sleeve-shaped region. In many cases, a single microcrystal reached the dividing line between the NCR and GCR, and could be used as a nucleus for the subsequent growth step which would take place within the GCR. After a nucleus has been formed, the perylene solution was further printed in the lower part of the GCR, to gradually fill the whole frame, thus initiating the growth of a single crystal at the desired location (Fig. 3c).

**Figure 4:**
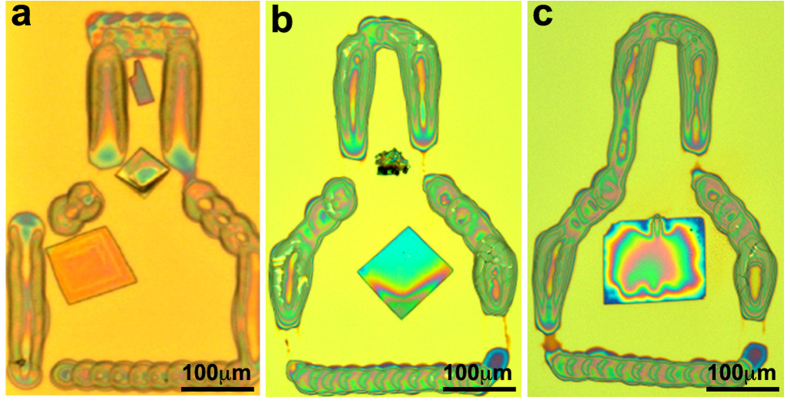
Three single crystals in AlCl_3_ confinement frames following their washing with isopropyl alcohol. (**a**) Two crystals grown, one at the NCR-GCR dividing line and one in the GCR. This was caused due to the drift of a nucleus from the NCR to the GCR during the growth step. (**b**) A Single crystal grown inside the AlCl_3_ confinement frame. Here, the excess nuclei are gathered in the NCR following the washing with isopropyl alcohol. (**c**) A single crystal, located in the center of the confinement frame due to its drift from the NCR-GCR dividing line during its growth. The excess nuclei in this case were washed away by the isopropyl alcohol.

**Figure 5:**
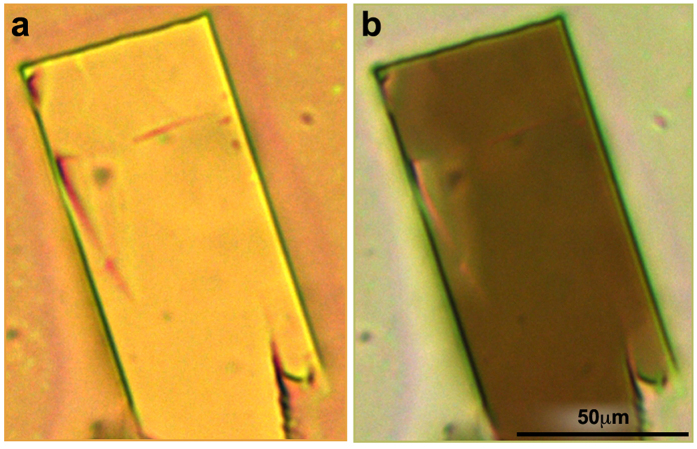
Microscope imaging of the single crystal in the confined frame on PET with two polarizers. Darkening occurred at a 50°.

During the process we have encountered several challenges. Occasionally, the nucleus was formed more than 30 μm above the NCR-GCR dividing line. In those cases, the deposited solution in the GCR did not reach the nucleus sufficiently fast, resulting in the formation of several microcrystals dispersed within the confinement. Moreover, in cases when the growth started farther up in the NCR, its strong directional influence resulted in formation of a long needle-like crystal connected to a rectangular crystal at its end (see Supplementary Fig. S2). Also, despite of the directional flow of the solution from the GCR to the NCR, in many cases a single crystal was detached from the NCR and drifted to other parts of the GCR (Fig. 4a and Supplementary Fig. S3). However, as long as no additional nuclei drifted away, a single crystal was indeed formed at the center of the frame (Fig. 4b,c). The high quality and uniformity of the single crystals were investigated by a polarized optical microscope; Fig. 5 demonstrates the uniform darkening of the crystal due to the rotation of the polarizing lens.

Following the printing and crystallization process, the samples were washed with cold isopropyl alcohol to remove the excess solvent (see Supplementary Fig. S2). As can be seen in Fig. 4b,c the excess of nuclei remained in the nucleation region, or were washed away by the isopropyl alcohol. Preliminary experiments showed that the deposited aluminum salt dissolves when exposed to water, however, at this stage we could not completely remove the frames without physically damaging the organic crystals. To determine the yield of the process, three independent printing experiments were conducted. In each experiment, an array of about 40 frames was printed. Following that, the perylene ink was printed to form single crystals (Supplementary Fig. S4), according to the procedure described in the experimental section. A success was considered as follows: obtaining only one single crystal, which is located in the growth control region, with a minimal top-side surface area of 2,500 μm^2^. The calculated yield of the process was 32 ± 8%.

### Device fabrication and characterization

**Figure 6:**
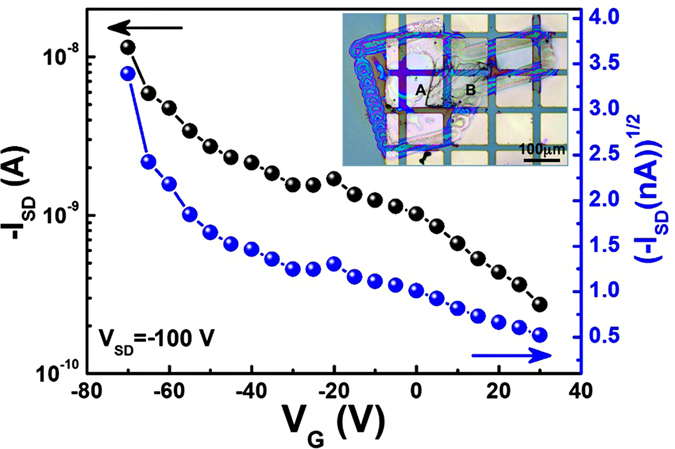
Transfer curve of individual perylene single crystal field-effect transistor. The inset is the optical image of the corresponding device, the channel is bridged between A and B.

The size of the printed perylene single crystal was typically ~100 μm x 100 μm (Fig. 4), which was suitable to make a field-effect transistor. Individual single-crystal perylene transistors have been made as shown in Fig. 6, in a top-contact, bottom-gate configuration. Copper grid was used as the mask for making the symmetrical electrodes (Au thin film, ~100 nm) by thermal evaporation, performed directly on the single crystals present within the frame. All transistors were fabricated and characterized in air, under ambient conditions. Typical p-type characteristic was observed in perylene single crystal field-effect transistor, having a mobility of ~4.0 × 10^–4^ cm^2^ V^−1^ s^−1^. This value is in the same range reported previously for perylene single crystal devices made by solution growth methods^28^.

It should be emphasized that the focus of the paper is to show for the first time that by using inkjet printing, which is a digital wet deposition process, we could induce crystallization at specific locations, without using any photolithographic processes. The process was demonstrated for an organic semiconductor, and therefore we performed a limited number of experiments on device fabrication. Making a high quality transistors was very challenging, due to factors such as residual solvents and the large thickness of the crystals (208 ± 80 nm) compared to those fabricated from vapor phase, which showed two or three orders of magnitude higher mobility^27^. Another possibility for the observed low transistor performance of the discussed device could be unintentional doping of the grown perylene crystal by hydrated AlCl_3_. Therefore, we performed energy dispersive X-ray analysis (EDXA) on four crystals to evaluate presence of Al or Cl (analysis performed on crystals close to and far from the frame). In all cases we could not find presence of Al and Cl, as shown in Supplementary Fig. S5. It is worth noticing that many polycrystalline perylene devices showed no field-effect activity at all.

## Conclusions

We have developed an all-additive manufacturing method for the controlled formation of a large number of individual single crystals of organic functional materials, at pre-designed locations. For the first time, we utilized the printer not only for solution deposition but also for the fabrication of a confining frame that enables the controlled growth of a single crystal within a specific region. This is the first step towards an all-inkjet printing process for production of single crystal-based devices. Moreover, since all processes take place under mild conditions, it will open a way for a variety of substrates and organic materials including semiconductors that could not be used until now. Currently, further work is being conducted to gain better control over the location of the single crystals, along with combining the controlled crystallization process with fabricating arrays of full devices including printed metallic electrodes. In addition, since the mobility of the printed crystals is lower than PVD grown crystals, further work should be done regarding controlling the crystal thickness and the selection of organic semiconducting materials with which the interaction of solvent residues will not reduce the mobility. The process described here presents a proof of concept for a wet deposition organic circuit technology in which individual steps have been developed and the assembly of these steps into a full production process is still required. It should be mentioned that by using a similar procedure described for printing on silicon wafers, we were able to grow single crystals also on PET, which is important for plastics electronics, although further optimization is required to increase the yield. We expect that by utilizing inkjet printing both for fabricating the crystallization vessel and for solution deposition, organic single crystal of various functional materials will become more accessible and their electronic and optical properties may be utilized in applications such as electronic papers or textiles.

## Methods

### Inkjet printing lithography of AlCl_3_ confinement frames

The ink was a 5%wt solution of AlCl_3_·6H_2_O (≥99%, Fluka) in a 1:1 mixture of dionized water and diethylene glycol butyl ether (≥99%, Aldrich). The substrates were polyethylene terephthalate, PET, (125 μm thickness, Jolybar Israel) and n-doped Silicon wafers with a 200 nm layer of SiO_2_ (Dashro Trade). The substrates were pre-cleaned by sonication, first in an ethanol (96%, Bio-lab) bath followed by an isopropyl alcohol (CP, Bio-Lab) bath and finally dried with a stream of N_2_. Inkjet printing of the confinement frames was carried out using an Omnijet100 printer (Unijet, Korea), with a Dimatix 1 picoliter printing head. **Printing on SiO_2_/Si**: The substrates temperature during printing was 60 °C. Printing was conducted at 800 Hz, and 1000 dpi. The waveform consisted of a 2 μs rise time to 30 Volts, another rise time of 6 μs to 40 volts and a 3 μs fall time to 0 volts. Each confinement frame consisted of 5 layers. **Printing on PET:** Same as on SiO_2_/Si with the following changes: The substrates temperature during printing was 65 °C. Printing was conducted at 500 Hz. Each confinement frame consisted of 10 printed layers. The thickness measurements were conducted by a Dektak 150 profiler (Veeco, USA). Elemental analysis was performed by using SEM microscope (XHR Magellan 400L) equipped with Energy-dispersive X-ray (EDX) probe (Oxford X-MAX, Oxford Instruments).

### Perylene crystal growth

The perylene ink was prepared by dissolving 5.6 mg of perylene (≥99.5%, Aldrich) in a mixture of 1 g of isophorone (98%, Acros) and 1 g of propylene glycol phenyl ether (≥93%, Aldrich). After 30 minutes of sonication, the saturated solution was left at room temperature overnight and then filtered. 1 mL of the ink was loaded into the cartridge with a 10 pL Dimatix printing head. **Printing on SiO_2_/Si**: The substrates temperature during printing was 35 °C. The printing head temperature was 27 °C. Printing of single drops was conducted at 1000 Hz. First, three drops were printed into the middle of the NCR, and the printed solution was kept for several minutes, to enable formation of the nucleus. Once a nucleus was formed, 100 drops were printed in the bottom third of the GCR, followed by two additional repetitions of 50 drops each at the same location. The solvent was allowed to evaporate at the same temperature and a single crystal was formed after a few minutes. Following the formation of the crystals, the substrate was gently washed with cold isopropyl alcohol and dried with N_2_. **Printing on PET:** Same as on SiO_2_/Si with the following changes: The substrates temperature during printing was 35 °C. First, nine drops were printed into the middle of the NCR. Once a sufficient nucleus formed, four repetitions of 50 drops were printed into the bottom third of the GCR.

### Device fabrication and characterization

Organic field-effect transistors based on the individual planar perylene single crystals were fabricated. A thin-bar copper grid was used as a mask for gold electrodes by thermal evaporation. The grid was directly put on the top surface of perylene single crystals and a scotch tape was used to stick the two surfaces together. Following that, the device was put into vacuum for thermal evaporation of gold thin films, which acted as the source and drain electrodes. The heavily-doped n-type silicon wafer was used as a gate electrode. *I-V* characteristics of perylene single-crystal FETs were measured using a Keithley 4200-SCS and a micromanipulator probe station in a shielded box at room temperature in air. To verify that the aluminum frame does not contribute to the charge transport property, blank measurements of the devices comprising solely the frame and the electrodes were conducted as well.

### Image acquisition

Photos of the printing and crystallization processes were taken with the built-in camera of the Omnijet 100 printer. Photos of the grown crystals were taken with a MicroPublisher 5.0 RTV camera (QImaging, Canada) on top of a CX41 optical microscope (Olympus).

## Additional Information

**How to cite this article**: Halevi, O. *et al*. Additive manufacturing of micrometric crystallization vessels and single crystals. *Sci. Rep.* **6**, 36786; doi: 10.1038/srep36786 (2016).

**Publisher’s note**: Springer Nature remains neutral with regard to jurisdictional claims in published maps and institutional affiliations.

## Supplementary Material

Supplementary Information
