# Supplementary material for: Additive manufacturing of micrometric crystallization vessels and single crystals
Source: Sci Rep. 2016 Nov 10;6:36786. doi: 10.1038/srep36786 (PMC5103199; doi:10.1038/srep36786)
Supplement: Supplementary Information [file srep36786-s1.pdf]

Supplementary information

Additive manufacturing of micrometric crystallization vessels and single crystals

Oded Halevi[1,2], Hui Jiang [2], Christian Kloc[2], Shlomo Magdassi[1*]*

[1] Casali Center of Applied Chemistry, Institute of Chemistry
The Hebrew University of Jerusalem Jerusalem, 91904, Israel,
[2] School of Material Science and Engineering
Nanyang Technological University, 639798, Singapore
* Correspondence to magdassi@mail.huji.ac.il

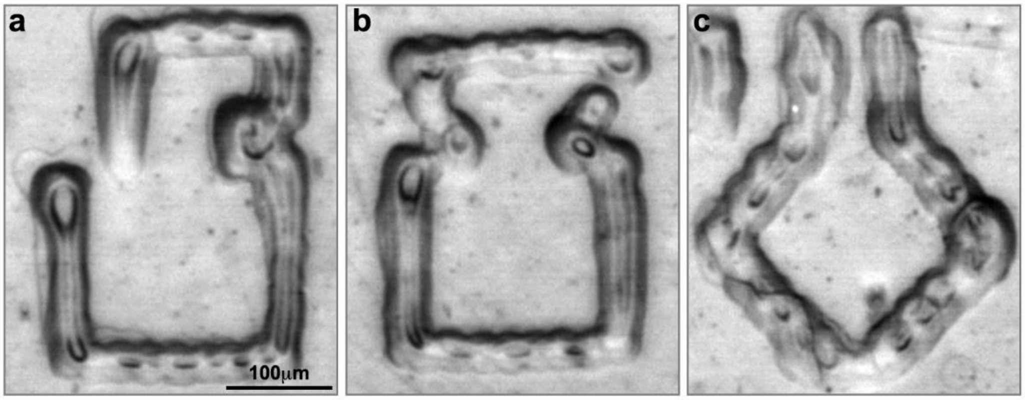

Supplementary Figure S1: Various geometries of confinement frames on PET.

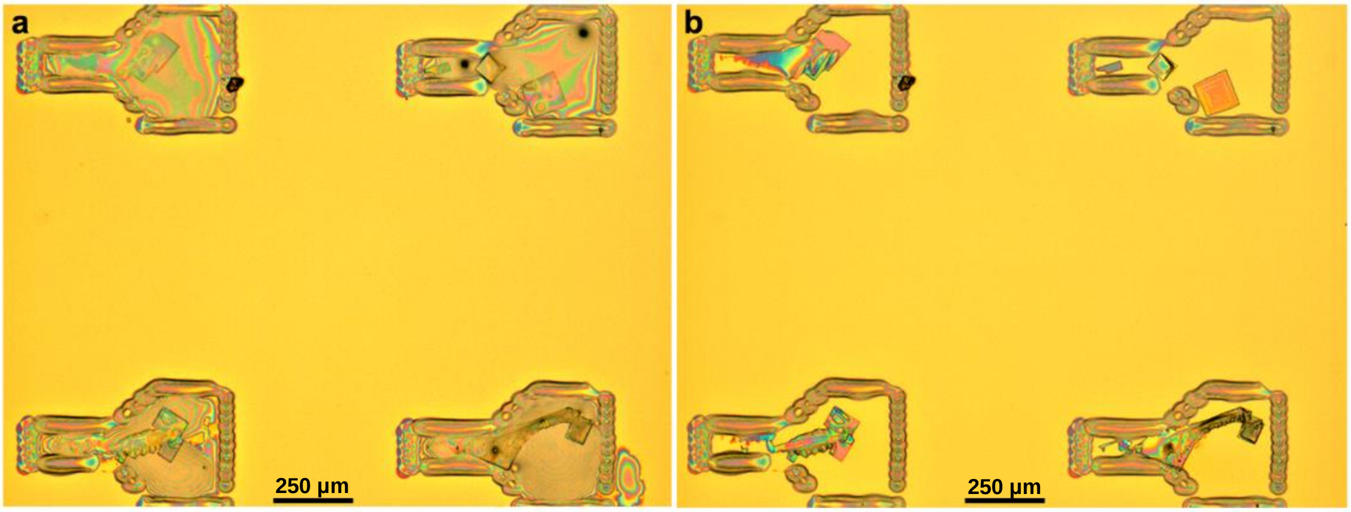

Supplementary Figure S2: An array of perylene crystals on Si/SiO_2 demonstrating the strong directional influence of the NCR on their growth. In the case of the two bottom frames, the growth commenced deep in the NCR, which resulted in a long needle-like crystal from which additional crystals were developed in the GCR. (**a**) Before washing with isopropyl alcohol. (**b**) After washing with isopropyl alcohol.

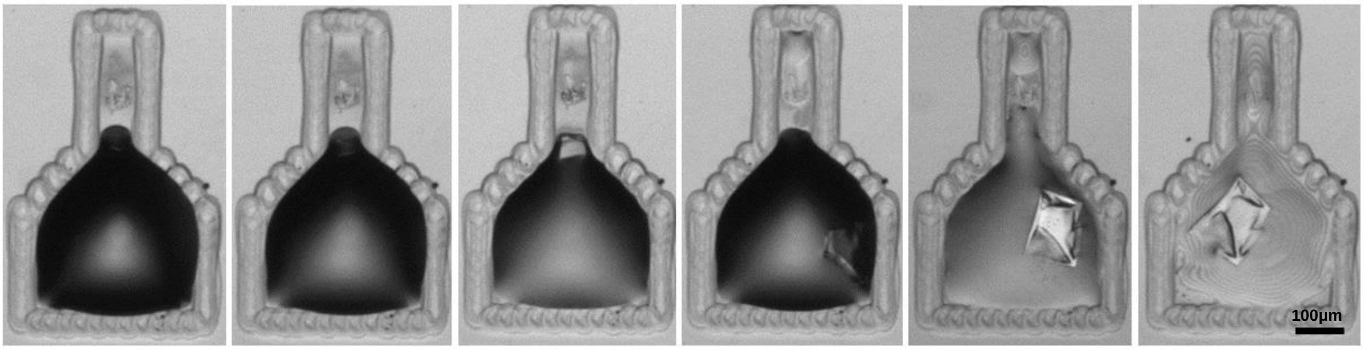

Supplementary Figure S3: The drift of a crystal from the NCR-GCR dividing line during growth.

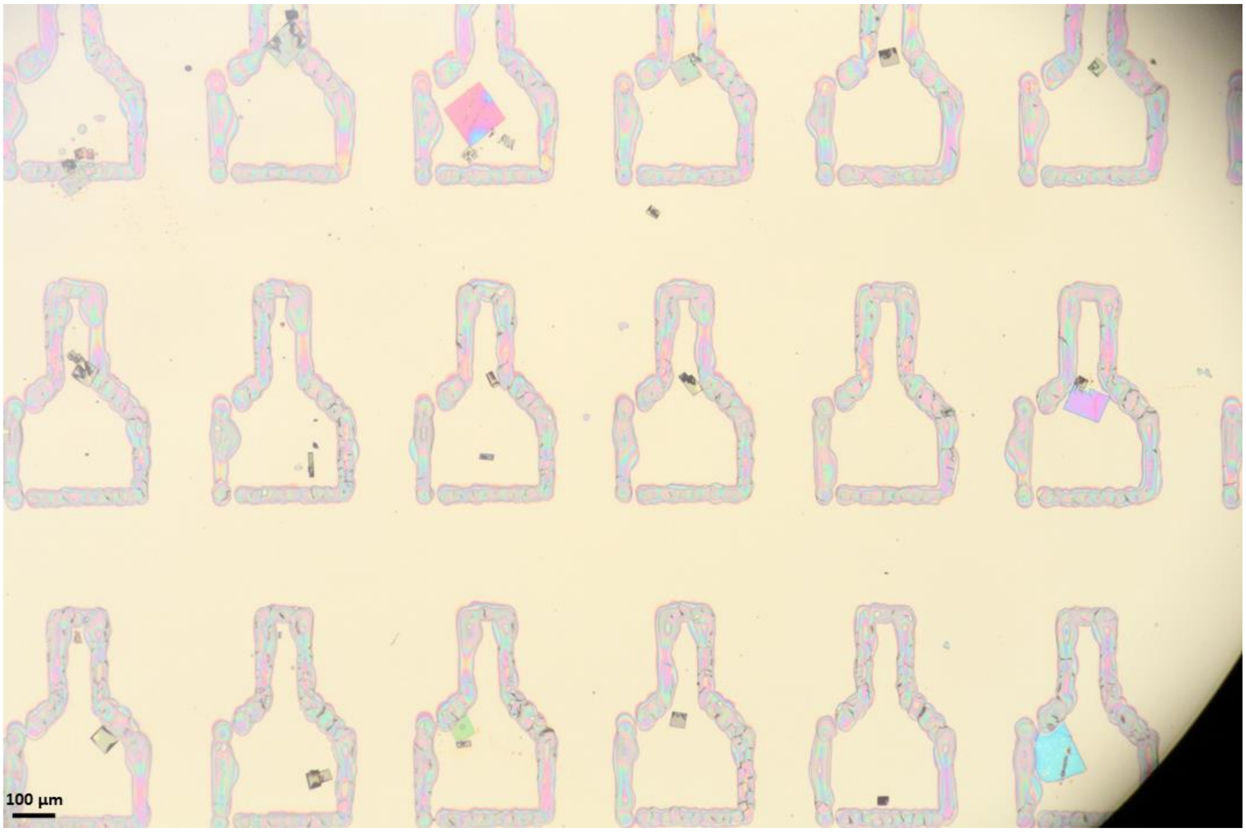

Supplementary Figure S4: An array of confinement frames with single crystals.

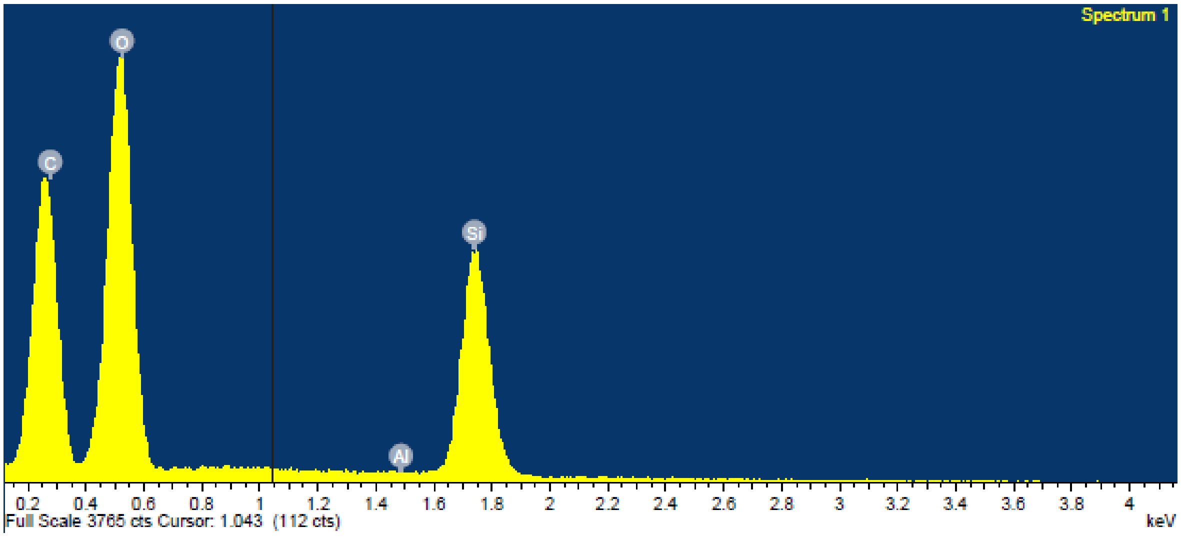

Spectrum processing :
No peaks omitted

Processing option : All elements analyzed (Normalised)
Number of iterations = 4

Standard :
C CaCO3 1-Jun-1999 12:00 AM
O SiO2 1-Jun-1999 12:00 AM
Al Al2O3 1-Jun-1999 12:00 AM
Si SiO2 1-Jun-1999 12:00 AM

Element	App Conc.	Intensity Corrn.	Weight%	Weight% Sigma	Atomic%
C K	297.27	1.3323	31.60	0.26	43.17
O K	569.90	2.1114	38.23	0.24	39.21
Al K	0.00	1.1686	0.00	0.00	0.00
Si K	232.76	1.0926	30.17	0.23	17.63
Totals			100.00		

Supplementary Figure S5: EDXA spectrum of perylene crystals inside an $AlCl_3$ frame.
